# Tribological Behaviour of Additively Manufactured Fiber-Reinforced Thermoplastic Composites in Various Environments

**DOI:** 10.3390/polym12071551

**Published:** 2020-07-13

**Authors:** Artur Prusinowski, Roman Kaczyński

**Affiliations:** Faculty of Mechanical Engineering, Bialystok University of Technology, 15-351 Bialystok, Poland; r.kaczynski@pb.edu.pl

**Keywords:** polymer-matrix composite, sliding wear, water environment, wear testing, additive manufacturing

## Abstract

Polymer composites with increased utility properties are becoming competition for conventional materials, in conjunction with additive manufacturing techniques. The aim of this study was to evaluate tribological characteristics of fibrous composites produced in fused deposition modeling (FDM) with the use of an innovative head with symmetrical feeding of the matrix material. Analysis of the influence of composite-forming temperature on their tribological properties allowed the determining of the optimal printing process parameters for this group of composites. Significant differences in the friction process of the same reinforced materials were observed in dry and wet environments. Fibrous composites showed 10 times lower wear intensity as well as two times lower friction value in water than in air. Research shows friction in the water environment ensures more even wear of the surface of the composites involved in the work. The article contains 3D microscopic imaging of the friction plane of the tested composites and a description of a typical course of material wear is described.

## 1. Introduction

Fused deposition modelling (FDM) is an additive manufacturing technology which involves layering of building material on a work surface until the desired element is created [1]. Dimensional accuracy is achieved at 50–100 microns [2]. In engineering applications, thermoplastic polymers are mainly used as the basic component [3]. The additional properties of manufactured elements are possible to achieve using composite materials [4].

There are three main methods that allow the use of materials reinforced with the FDM. One such method is the addition of a filament of short fibers or metallic particles at the molding level to the screw extruder [5,6]. Extruded composite materials with a reinforcement content between 1% to 10% were subjected to a tensile strength test, resulting in an 18% increase in strength [7]. Over twofold increase in tensile strength of fiberglass-reinforced polypropylene composite was obtained with a 30% content of fillers for unreinforced material, with a simultaneous decrease in the elongation of the stretched sample [8].

The second method for producing an element from a composite material used in FDM is the use of prefabricated continuous fiber material [9]. The research conducted on the impact of the percentage content of fiber fillers in composites used in a given molding method showed a correlation between an increase in mechanical strength and increase in the amount of fillers [10,11,12].

The last group of composite-forming methods in the FDM involves the feeding of continuous reinforcement directly to the extrusion region of the head composite separately to supplying matrix material to the head. The research that focused on the tensile strength of the composites showed a significant increase in tensile strength as well as a change in the tensile nature of the materials from plastic-elastic to elastic [13,14,15].

Numerical simulations of heat distribution in the extrusion head of a composite material used in FDM allowed the development of the optimal construction of the entire system [16]. Analysis of polymer-polymer composite extrusion made it possible to determine an adequate work program for plastic feeders and confirmed the results of the numerical simulations [17].

Numerous studies on the impact of FDM parameters on mechanical (tensile strength, compression, bending and fatigue strength) and tribological properties of test samples for various categories of materials and different sets of process parameters/production conditions were conducted [18,19,20,21]. The influence of the printing layer height, model orientation, model filling and other parameters on the microscopic appearance of the friction surfaces of the examined details was established [22,23]. The tests carried out on the impact of coloring the same type of material on the mechanical properties did not show significant changes in the parameters of these materials [24,25].

The influence of reinforcement and matrix parameters of polymer composites manufactured by standard methods on their mechanical and tribological properties has been widely studied [26,27,28,29]. Research on tribological wear of the polyacryl ether ketone matrix composite and two types of reinforcements: short glass fibers and synthetic graphite at different loads and friction speeds showed an increase in abrasive wear of materials along with an increase in friction speed regardless of the used composite reinforcements [30,31].

This study presents the results on tribological properties (taking into account the various working environments) of fiber composites produced in the proprietary FDM extrusion head with direct feeding of reinforcing fiber and symmetrical feeding of the matrix material.

## 2. Materials and Methods

### 2.1. Method of Forming Polymer Fibrous Composites

**Figure 1:**
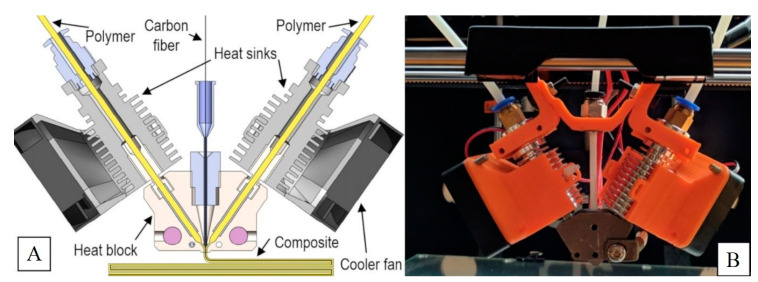
(**A**) Diagram of the extrusion head with symmetrical feeding of matrix material and (**B**) the made prototype.

Polymer fibrous composites used in the research part of the study were produced using a patented extrusion head mounted on a 3D printer working in FDM technology. Figure 1A presents a scheme for forming a thermoplastic fiber composite in a patented extrusion head with symmetrical feeding of matrix material. The matrix material in the form of a wire with a diameter of 1.75 mm was fed through two transport channels. The continuous carbon fiber was fed to the die head along the material extrusion axis where the composite was formed. The reinforced material was extruded in layers into the working field of the device to form a fabricated detail. The completed prototype of the head is shown in Figure 1B.

### 2.2. Materials

In the presented study, a matrix material of acrylonitrile-butadiene-styrene (ABS) was used in the form of a filament with reduced linear shrinkage. The polymer density was 1.05 g/cm^3^ and thermal conductivity was 0.1 W/m∙K. The composite filler was the continuous carbon fibers of the Japanese company Torayca T300-1000 66 TEX (Toray Industries Inc., Tokyo, Japan) in the form of a 1000-fiber bundle with a tensile strength of 3530 MPa at 1.5% elongation. The fiber mass density was 1.76 g/cm^3^ and thermal conductivity was 25 W/m∙K. Steel of 40 HM was used as the disc material in the tribological tests, which has a yield strength of 880 MPa, increased abrasion resistance and can be easily modified by mechanical or heat treatment.

**Figure 2:**
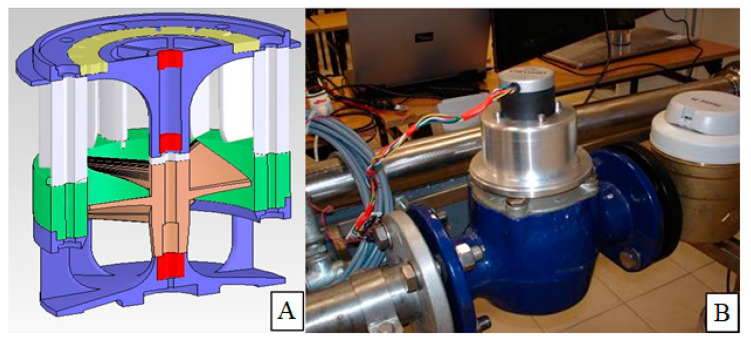
Cross-section of (**A**) a mini generator model with sliding sleeves marked (in red), (**B**) the real device.

Tests on these materials constituted the basis of part of the work in the special purpose project 6 ZR6 2009C/07296 on construction of a mini-energy generator including the flow of the working factor as a working environment. Figure 2A shows the cross-section of the turbine, while Figure 2B shows the complete mini generator during testing. Analysis of the friction of the material system in various working environments and at different loads allowed the production of composite sliding sleeves reinforced with carbon fiber, necessary to embed the rotor in the turbine body of the electricity mini-generator. In this application, the smallest weight loss of the sliding elements is crucial. Tribological test parameters of this work—pressure and sliding speeds—were selected based on the operating conditions of the device. The selection of pressure and speed was implemented in two stages. First, measurements of the rotational speed and moment occurring in the mini generator were made, and preliminary tests were carried out at v = const (first series of tests) and at *p* = const (second series).

### 2.3. The Method of Estimating the Effective Content of Reinforcements

The effective content of continuous fibers reinforcing formed polymer composites was estimated based on the developed formula taking into account FDM process parameters—Equation (1):

(1) $$W_{F}=\frac{\sum_{i=1}^{N}L_{\mathrm{Fi}}\times d^{2}\times \pi \times f\times \sin\left(\alpha \right)}{\sum_{i=1}^{N}L_{\mathrm{Ci}}\times 4\times h\times s}$$

where: *h*—height of a single layer, *s*—pathwidth, L_F__i_—number of reinforced paths in the layer, L_Ci_—total number of paths in the layer, *N*—number of model layers, *d*—diameter of the reinforcing fiber, *f*—amount of fibers in a single path, sin(α)—deviation of the fiber axis from the reference plane.

**Figure 3:**
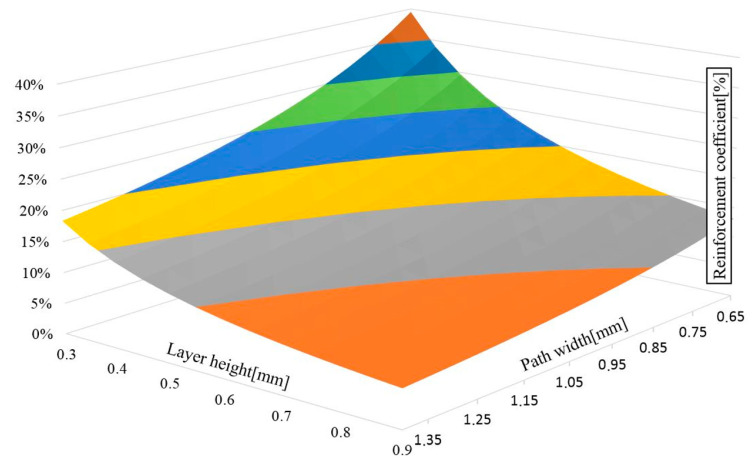
The effect of path height and width on the fiber content factor.

The developed formula enables the determination of the effect of track height and width on the content factor of the reinforcement. Figure 3 presents a graph based on calculations in which a fixed number of layers and paths in the model, a constant angle of deviation of the composite paths as well as an equal amount of reinforcing material, were adopted.

Anisotropicity of composites has been included. The calculated effective fiber reinforcement value is prospectively a more accurate method of comparing composites than the one based on the matrix/reinforcement volume ratio throughout the composite without specifying the orientation. The formulas presented can be easily used in calculation programs and supporting 3D printing.

### 2.4. Tribological Studies of the Composite

**Figure 4:**
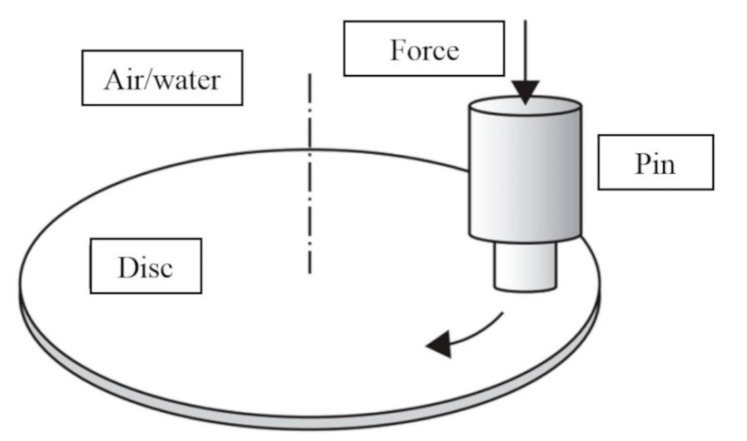
The concept of tribological examination in the pin−disc friction system.

Tribological tests of the formed composites were carried out in the sliding test system pin−disc, see diagram in Figure 4. In experiments, a pin made of fiber composite with a diameter of 6 mm and height of 10 mm was pressed with a predetermined force against a rotating counter-sample made of 40 HM steel. Depending on the type, the tests were performed in a dry environment and in water.

**Figure 5:**
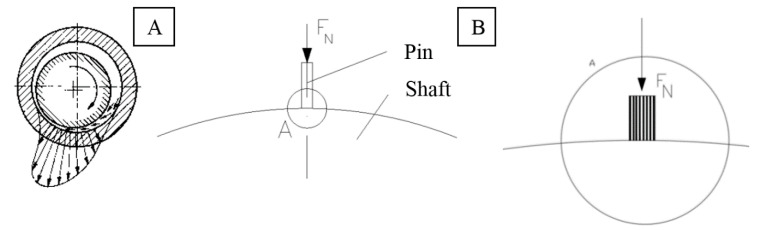
(**A**) Force distribution in a slide bearing, (**B**) contact: composite sample—shaft end, detail—distributed contact of composite sample and shaft end.

The carbon fibers were placed in the samples perpendicular to the direction of friction, due to the optimal tribological characteristics of this type of material. The study was based on a flat friction contact model (surface−surface), which reflects the contact of both components (sleeves and shaft) in the bearing (Figure 5A). Due to the small dimensions of the carbon fibers (fiber diameter is 7 µm), it was assumed that the surface of the shaft neck with a much larger radius can be roughly treated as a plane (Figure 5B).

Parameters of composites formed as part of the first stage of research, determining the influence of the temperature of forming composites on their tribological properties are summarized in Table 1. The aim of the second stage of the study was to determine the impact of the magnitude of the load as well as the working environment on the tribological properties of the composite materials (a list of material parameters is provided in Table 2).

The possibility of comparing the quantitative linear wear and mass loss of the tested composites was obtained by using two factors: the linear wear intensity, L_WI_ (Equation (2)) and the mass wear intensity, M_WI_ (Equation (3)).

(2) $$L_{\mathrm{WI}}=\frac{∆L}{s\times P_{s}}$$

(3) $$M_{\mathrm{WI}}=\frac{∆M}{s\times P_{s}}$$

where: ∆L—change in sample height after the test, ∆M—change in sample weight after the test *s*—friction path, P_s_—initial sample surface area.

Microscopic photos of the friction surfaces of the examined materials were taken using the LEXT OLS4000 confocal microscope (Bialystok University of Technology, Bialystok, Poland), which allows for total image magnification up to 17.280 times. The mass loss measurement of the samples after tribological tests was measured with an accuracy of d = 0.01 mg using a RADWAG XA 210.4Y PLUS device (Bialystok University of Technology, Bialystok, Poland).

## 3. Results and Discussion

### 3.1. Modifying the Geometrical Properties of Composites

**Figure 6:**
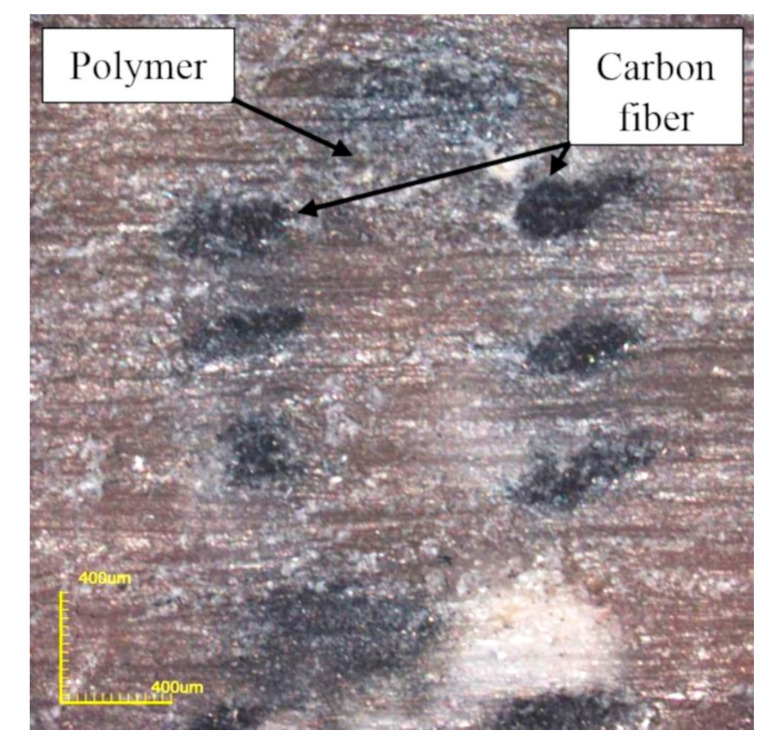
Cross-section of composite across bundles of reinforcing fibers.

The first stage of research was aimed at determining the possibilities of modifying the geometric properties of formed composites. The simplicity of changing the composite volume filling factor was crucial for this method of their production. Microscopic images of cross-sections of extruded materials were made (Figure 6) and measurements of the distance of subsequent fiber bundles in two planes were performed: within one layer of the material and between layers.

**Figure 7:**
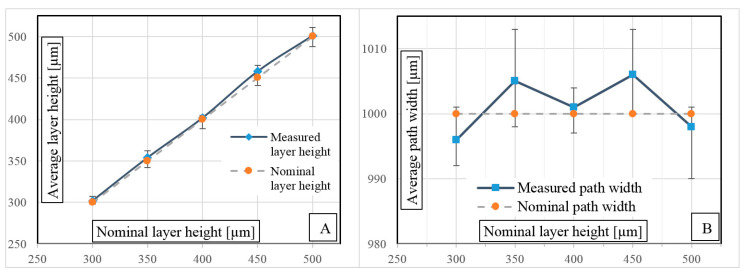
Comparison of measured and nominal (**A**) composite print layer heights and (**B**) single path widths.

Figure 7 shows the results of the microscopic examination. The difference in measured values of the height of the printed layer (Figure 7A) compared to the nominal, programmed results was 0.5–1.5%. The width of a single composite path (Figure 7B) depending on the height of the printing layer ranged from 990 to 1012 µm in the tested range.

It was possible to determine the influence of FDM process variables on the geometrical properties of obtained ABS/CF composites. The ability to change the volume filling of composites by providing a constant amount of reinforcing fiber and simplifying the control of the 3D printing device was determined.

### 3.2. The Influence of Composite Forming Temperature on Tribological Properties

Part of the research analyses the correlation between the composite forming temperature in the 3D printing head and its tribological properties. Table 3 presents the plan for the first part of the experiment. One percent filler content and one pressure generated per sample were selected. Composite extrusion temperatures between 225 and 245 °C were used, which corresponds to the range of suggested printing temperatures for this matrix polymer. Tests of composites formed at particular temperatures were repeated five times to confirm the repeatability of the experimental results.

The results of tribological tests on ABS/carbon fiber composites-40 HM steel enabled comparative analysis of composites with different forming temperatures in the head of a 3D printing device. In the present study, the most important features for choosing the forming temperature for composites were the low value of mass wear intensity and the appearance of the friction surface after the test.

**Figure 8:**
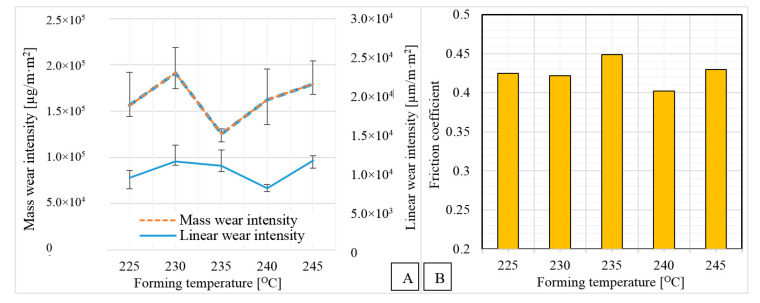
(**A**) Intensity of mass and linear wear, (**B**) coefficient of friction at different composite forming temperatures.

Figure 8A shows the mass and linear wear intensities of samples of the ABS CF9.4 composite formed at temperatures in the range 225–245 °C. The lowest calculated value of mass wear occurred with the composite formed at 235 °C–1.25 × 10^5^ µg/m·m^2^, while the highest at 230 °C–1.91 × 10^5^ + 05 µg/m·m^2^. The difference in wear intensity between these composites was 6.6 × 10^4^ µg/m·m^2^. The lowest value of the linear wear was characterized by a composite formed at a temperature of 240 °C–8.01 × 10^3^ µm/m·m^2^, while the highest wear was shown by the composite formed at 230 °C–1.15 × 10^4^ µm/m·m^2^. Figure 8B shows a summary of the calculated friction coefficients in fiber composites depending on the forming temperature.

The material formed at temperature 240 °C had the lowest coefficient of friction. All coefficients were in the range of 0.4–0.45. In terms of the parameters tested, a simple relationship between forming temperature and tribological properties was not visible. This was due to the formation of composites in a narrow range of temperatures close to optimal for ABS in the FDM technique.

**Figure 9:**
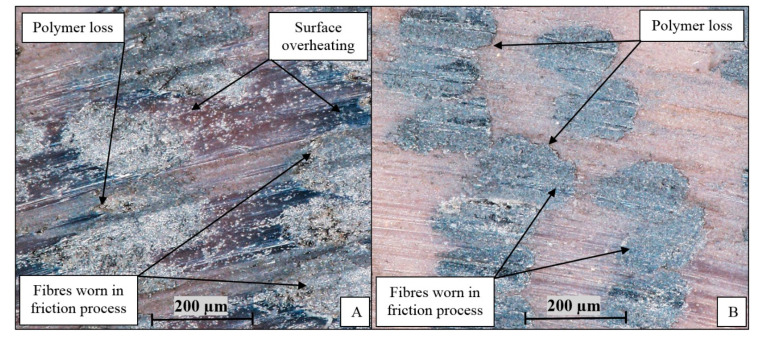
The surface after testing the tribological wear of the composite formed at temperature: (**A**) 225 °C, (**B**) 235 °C.

Despite obtaining comparable results of mass/linear wear intensity and friction coefficients of friction systems, differences in friction surfaces of composites are noticeable. In the case of material formed at 225 °C, overheating of the friction surface, numerous wear products in the form of parts of fibers embedded in the matrix and polymer breakouts at the ABS/fiber connection were observed (Figure 9A). The composite formed at 235 °C (Figure 9B) had a smaller amount of used fiber parts and no overheating of the matrix polymer was observed. Considering the intensities of mass wear, friction coefficients and microscopic images of the friction surface after tests, 235 °C was the appropriate forming temperature for this group of composites. In the following stages of research, the composites were made at this temperature.

### 3.3. Impact of Unit Pressure on Wear Intensity and Friction Coefficient

In order to properly examine the properties of fiber composites, it was necessary to know the effect of pressure on the kinematic friction coefficient of the system and the intensity of linear and mass wear of materials. The experimental parameters are shown in Table 4. Experiments on composites with specific reinforcement contents were repeated five times to confirm the repeatability of the test results.

**Figure 10:**
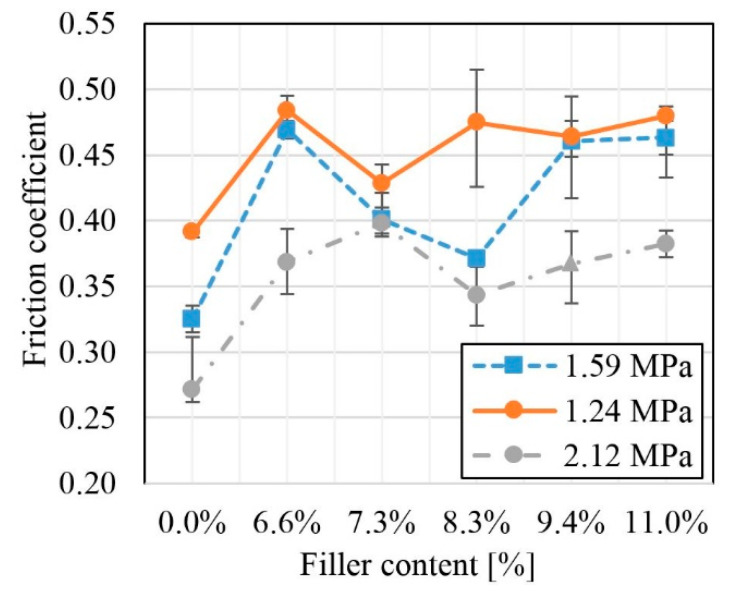
The kinetic friction coefficient depending on the unit pressures.

Based on the friction force diagrams, the values of the friction coefficients in the systems were determined depending on the adopted unit pressures. Figure 10 presents graphs of calculated values of friction coefficients for individual systems at selected unit pressures. As the pressure increased, the system’s friction coefficient decreased. Regardless of the pressure, the lowest values of the kinematic friction coefficient were achieved by the material not reinforced with carbon fiber. This was due to the uniform application of the polymer layer to the steel counter-sample and simultaneous reduction of roughness. The adhesion between the layer of applied material and the polymer surface was significantly smaller than at the metal−polymer interface. The presence of reinforcement reduced the contact surfaces of uniform polymers by breaking the applied layer.

**Figure 11:**
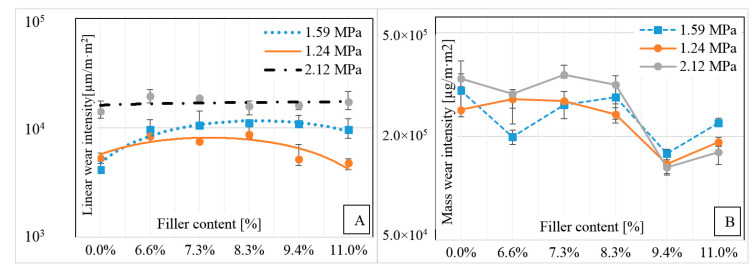
Influence of reinforcing fiber content on (**A**) linear wear intensity, (**B**) mass wear intensity.

Tribological studies of formed composites showed significant differences in the intensity of linear wear (Figure 11A) and mass wear (Figure 11B) of the given samples. Along with the increase in pressure, the intensity of linear wear increased. At pressures 1.24 and 1.59 MPa there was a decrease in wear between the samples with the smallest and highest degree of strengthening. Samples working under a pressure of 2.12 MPa did not show significant differences in wear depending on the degree of composite reinforcement. This was due to the increased temperature of the entire friction system. Carbon fibers bent under heavy load were fused to the matrix polymer that exceeded the softening point on the surface.

The mass wear intensity curve in all cases was convergent in terms of mileage, slightly changing in value. The fiber volume content above 9% allowed lower wear intensity compared to the unreinforced material. The unreinforced material showed wear mass intensity with values close to the maximum measured in this experiment. The composite with 9.43% reinforcements showed better tribological properties than other tested materials, especially the mass wear intensity.

### 3.4. Impact of Work Environment on the Tribological Properties of Composites

The research aims to determine the impact of the work environment on the tribological properties of developed composite materials. The experiment was carried out in two types of environment: in air and water. Table 5 contains detailed data on the second stage of tribological studies. The tests were carried out on composites with different contents of reinforcing fibers at a constant load value of 1.59 MPa, and sliding speed of 1.0 m/s. The experiment was repeated five times for each layer height and work environment to confirm the repeatability of the results.

**Figure 12:**
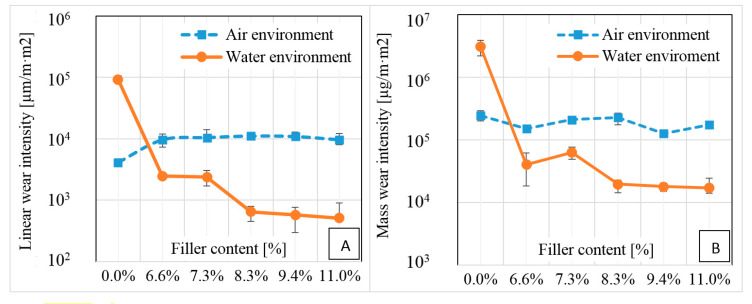
Influence of composite reinforcement on (**A**) intensity of linear wear, (**B**) intensity of mass wear in various environments of the system.

A comparison of the relationship between the composite reinforcement with carbon fibers and the intensity of linear and mass wear in the case of dry friction and in a water environment indicates a large difference in the observed phenomena (Figure 12). In both cases, the material not reinforced with fibers showed a greater intensity of wear in the water environment than in air.

Materials reinforced with carbon fiber in a dry environment maintain the intensity of linear wear between 9.64 × 10^3^ and 1.42 × 10^4^ µm/m·m^2^. In the water environment, the dependence of linear and mass wear intensities on the percentage content of the filler in the composite was observed. The linear wear intensity in the water environment was an order of magnitude lower than in the case of dry friction. The intensity of mass wear for the tested fiber composites was significantly higher in the case of dry friction than in the water environment and was in the range 1.25 × 10^5^–2.65 × 10^5^ µg/m·m^2^. In water, the intensity of mass wear was 6.25 × 10^4^–1.71 × 10^4^ µg/m·m^2^, which was many times less in comparison to the dry environment. An increase in the amount of carbon fiber between 7.3% and 8.3% in volume causes a decrease in the consumption of composites. It was observed that the fiber content above 8.3% did not significantly affect the examined tribological properties.

**Figure 13:**
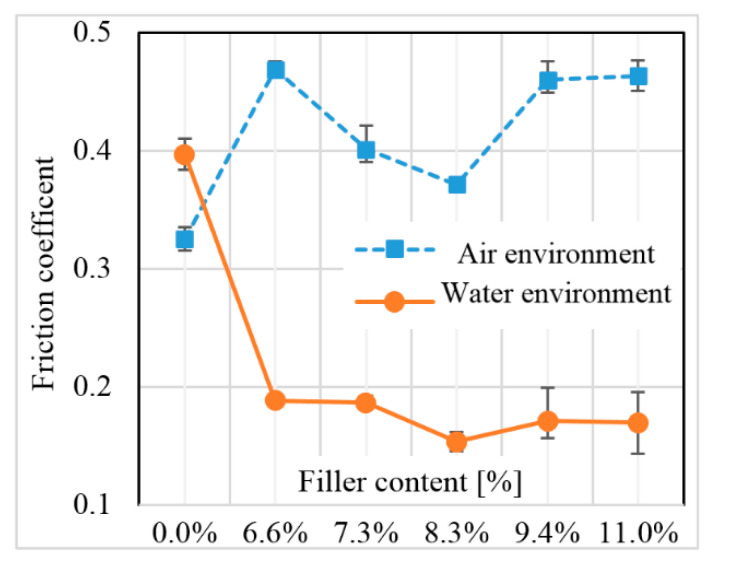
Influence of composite reinforcement on the kinematic friction coefficient in various environments.

The friction coefficient of the system has a significant impact when selecting tribologically loaded materials. In the case of an unreinforced sample, the coefficient of friction in water was higher than in a dry environment (Figure 13). Without water, a large part of the matrix material was applied to the steel counter-sample, reducing the coefficient of friction in the system. When testing a friction node in water, part of the worn product was immediately lifted from the immediate vicinity of the friction surface, thereby reducing the amount of polymer deposited from increasing the friction coefficient.

**Figure 14:**
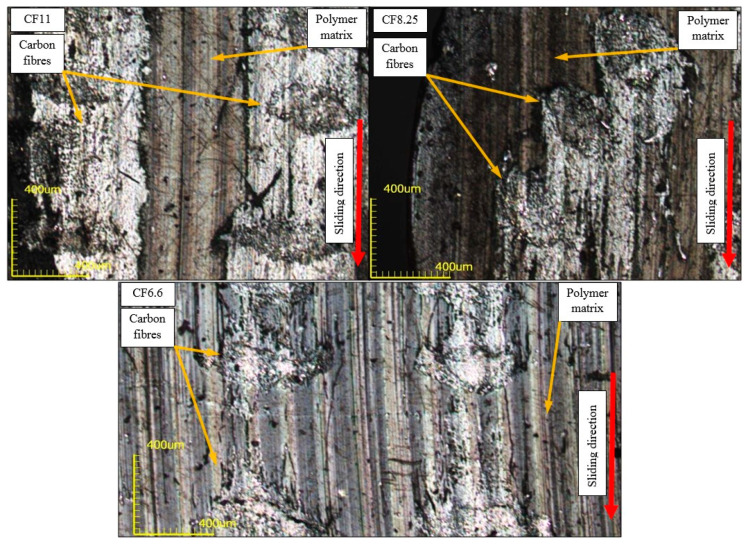
Surfaces of acrylonitrile-butadiene-styrene (ABS) CF11; CF8.25; CF6.6 composites after wear testing in a water environment.

In the case of tested fiber composites in a water environment, carbon fibers played a major role in the friction process, significantly reducing the kinematic friction coefficient. Water lowers the overall temperature of the entire system and additionally lubricates the contact surfaces of friction steam so that carbon fibers do not melt into the plasticized matrix material as in dry friction. A slight decrease in the coefficient of friction can be observed associated with the increase in the number of fibers directly involved in the friction process. The surface wear of fiber composites with different degrees of reinforcement at the same pressure values and sliding speeds was significantly different, as shown in Figure 14.

**Figure 15:**
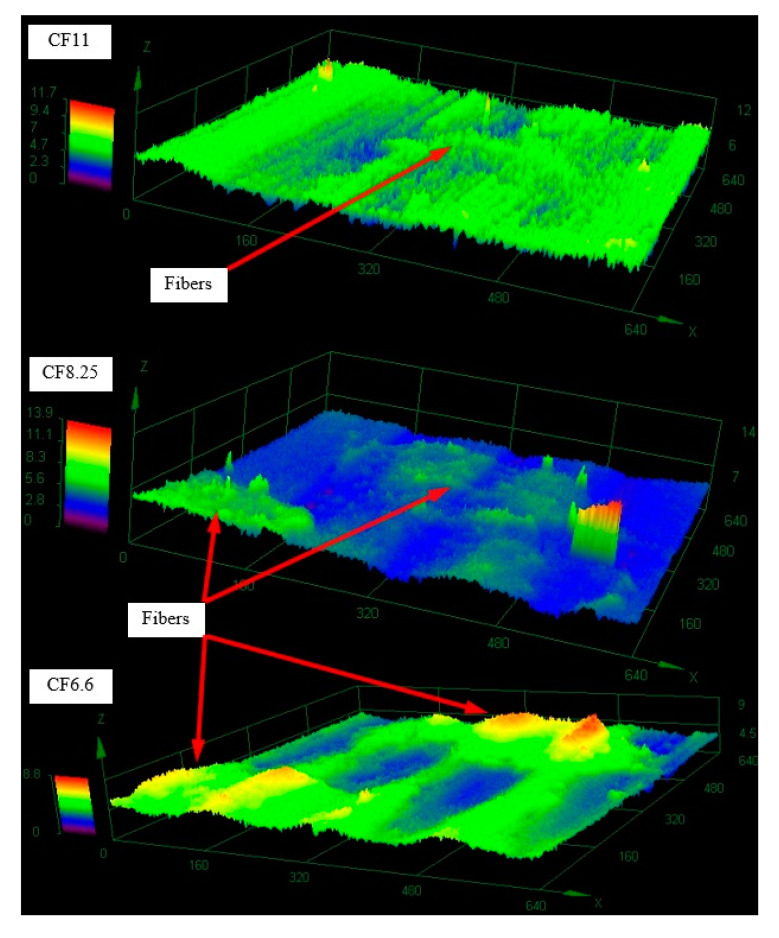
Topographies of ABS CF11; CF8.25; CF6.6 composites after tribological studies.

The number of carbon fibers perpendicular to the friction surface had a significant impact on the operating conditions of the friction system. At low reinforcements (6.6%) the load was transferred by a relatively small amount of fibers, whose deformation caused increased matrix wear. Some of them were permanently embedded in the polymer during friction, while most of them returned to their original shape after the disappearance of the load, which was visible on the topographic scan (Figure 15). As the content of carbon fiber bundles increased from 8.25%, the load was distributed over a larger number of reinforcements resulting in a change in the nature of surface wear. Part of the used material was again deposited on the friction surface behind the fiber bundles (looking in the direction of friction) creating secondary structures visible in the microscopic images as lighter fields. The produced particles of materials were characterized by increased strength, compared to the matrix, due to the presence of nondirected carbon microfibers. Figure 15 shows the surface of these materials after testing, more uniform than samples with a lower degree of reinforcement. The larger contact surface of the actual composite/steel association translated into lower intensity of linear and mass wear.

**Figure 16:**
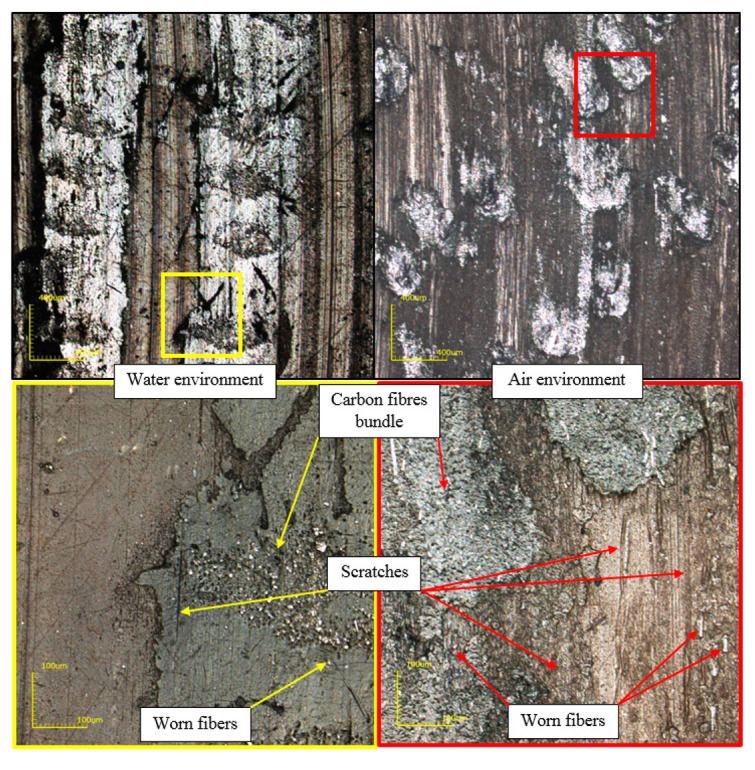
Comparison of friction surfaces of composites tested in water and air.

The operating environment of the friction system played a key role in the phenomena occurring on the surface of fibrous composites in the friction process. Microscopic images (Figure 16) showed significantly less detached carbon fiber from the main beam in a water environment than in dry friction. Products of wear after detachment from the test material were discharged with the water, which resulted in fewer scratches on the friction surface.

**Figure 17:**
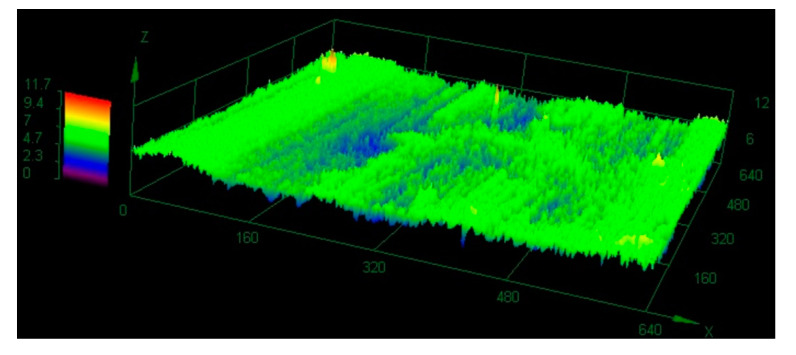
The 3D mapping of the friction surface of the composite tested in the water environment.

**Figure 18:**
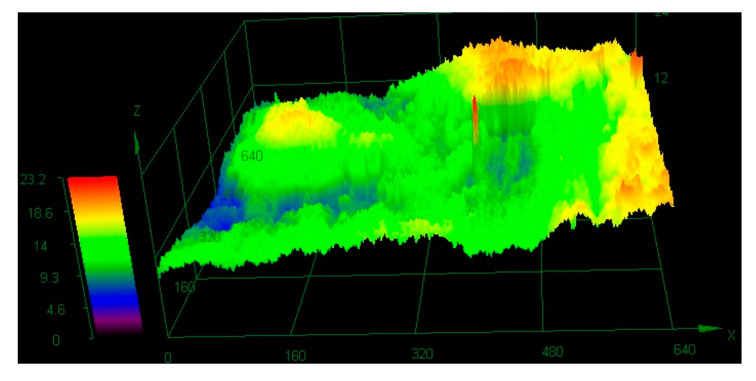
The 3D mapping of the friction surface of the composite tested in a dry environment.

Unevenness occurring on the friction surfaces of composites in both environments was dependent on the presence of reinforcing fibers. The surface of the material working in the water (Figure 17) was evenly lapped, and the height amplitude practically two times smaller than in dry friction: 11.7 µm. In the case of dry friction (Figure 18) the carbon fibers protruded significantly out of the polymer plane. The matrix material was pulled out during friction and the load was transferred by the reinforcement. The height amplitude in the study area was 23.2 µm.

By analyzing the durability of the tested materials, friction in the water environment ensures more even wear of composites while reducing the intensity of wear and the coefficient of friction.

**Figure 19:**
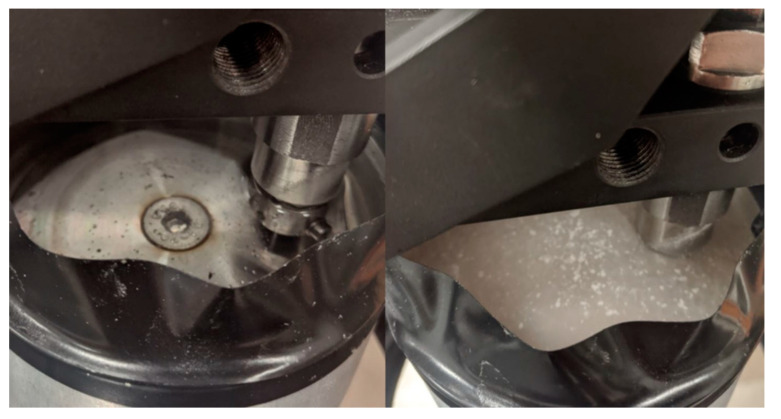
(**Left**) Water contamination with wear products after examination of the fibrous composite and (**right**) the unreinforced ABS polymer.

Figure 19 presents photos of the pin−disc−water system after tribological tests. On the left side there are a few parts of the composite ripped out of the test pin during the friction process. Part of the polymer was applied to a steel counter-sample, while carbon fiber particles moved loosely in water. On the counter-sample used for tribological studies of the fiber composite, in addition to the polymer, carbon fiber particles were deposited on the outer edge of the friction trail. The used fibers were pushed out of the friction pair contact surface through the water flowing around the system and the momentum of the slide.

In the case of a sample made only of ABS polymer, the wear of the pin was higher than in the case of dry friction. Analyzing the use of this type of material for tribological applications in a water environment, showed that a large number of products of wear in water can be dangerous for the entire system of the device and did not allow for adequate life of the friction node.

## 4. Conclusions

In this study, we examined the tribological properties of carbon-fiber reinforced thermoplastic composites formed using the additive printing technique. In addition, the effects of changing process parameters on the geometrical properties of fiber composites were determined. The use of an innovative extrusion head and research into the physical properties of formed composites allow the following conclusions to be drawn:

The presented method of estimating the effective content of reinforcements allows the determination of the effect of path height and width on the fiber content factor.

Analysis of the influence of the composite-forming temperature on their tribological properties established the optimal printing process parameters for this group of composites.

Studies of the tribological properties of composites in a dry condition revealed significant differences in the intensity of linear and mass wear of the tested materials. Fiber volume content above 9% allows lower wear intensity compared to the unreinforced material.

Significant differences were observed in the friction process occurring in dry and water environments of the same reinforced materials. Fibrous composites showed 10 times less wear intensity and a twofold lower friction coefficient in water than in air.

The tribological system without fiber reinforcement results in a substantial increase in material wear regardless of the environment, which undermines the use of such associations. It is only through the controlled addition of reinforcing fibers that the operating conditions of the system change and significantly increase its life span.

## Figures and Tables

**Table 1:** Parameters of fibrous composites, 1st stage of research.

No.	Layer Height μm	Forming Temperature °C	Filler Content %*v*/*v*
1		225	
2		230	
3	350	235	9.43
4		240	
5		245	

**Table 2:** Parameters of formed fiber composites, 2nd stage of research.

No.	Layer Height μm	Forming Temperature °C	Filler Content %*v*/*v*
1	300		11
2	350		9.43
3	400	235	8.25
4	450		7.33
5	500		6.6

**Table 3:** Experiment parameters I.

Filler Content %*v*/*v*	Layer Height μm	Forming Temperature °C	Pressure MPa	Sliding Speed m/s	Environment
		225 230 235 240 245	1.59	1.0	Air
	
9.43	350				
	
	
	

**Table 4:** Experiment parameters II.

Filler Content %*v*/*v*	Layer Height μm	Forming Temperature °C	Pressure MPa	Sliding Speed m/s	Environment
0.00	300	235	1.24 1.59 2.12	1.0	Air
11.0	300				
9.43	350				
8.25	400				
7.33	450				
6.60	500				

**Table 5:** Experiment parameters III.

Filler Content %*v*/*v*	Layer Height μm	Forming Temperature °C	Pressure, MPa	Sliding Speed m/s	Environment
0.00	300	235	1.59	1.0	Air Water
11.0	300				
9.43	350				
8.25	400				
7.33	450				
6.60	500				
